# Effect of Edible and Active Coating (with Rosemary and Oregano Essential Oils) on Beef Characteristics and Consumer Acceptability

**DOI:** 10.1371/journal.pone.0160535

**Published:** 2016-08-09

**Authors:** Ana Carolina Pelaes Vital, Ana Guerrero, Jessica de Oliveira Monteschio, Maribel Velandia Valero, Camila Barbosa Carvalho, Benício Alves de Abreu Filho, Grasiele Scaramal Madrona, Ivanor Nunes do Prado

**Affiliations:** 1 Food Science Post-Graduate Program, Universidade Estadual de Maringá, Maringá, Brazil; 2 Animal Science Department, Universidade Estadual de Maringá, Maringá, Brazil; 3 Basic Health Science Department, Universidade Estadual de Maringá, Maringá, Brazil; 4 Food Engineering Department, Universidade Estadual de Maringá, Maringá, Brazil; 5 Animal Science Department, Universidade Estadual de Maringá, Maringá, Brazil; Islamic Azad University Mashhad Branch, ISLAMIC REPUBLIC OF IRAN

## Abstract

The effects of an alginate-based edible coating containing natural antioxidants (rosemary and oregano essential oils) on lipid oxidation, color preservation, water losses, texture and pH of beef steaks during 14 days of display were studied. The essential oil, edible coating and beef antioxidant activities, and beef consumer acceptability were also investigated. The edible coatings decreased lipid oxidation of the meat compared to the control. The coating with oregano was most effective (46.81% decrease in lipid oxidation) and also showed the highest antioxidant activity. The coatings significantly decreased color losses, water losses and shear force compared to the control. The coatings had a significant effect on consumer perception of odor, flavor and overall acceptance of the beef. In particular, the oregano coating showed significantly high values (approximately 7 in a 9-point scale). Active edible coatings containing natural antioxidants could improve meat product stability and therefore have potential use in the food industry.

## 1. Introduction

Beef is among the most consumed meats worldwide. The meat has a high protein content and an abundance of minerals, vitamins and fatty acids [1]. Consumers associate several meat attributes with freshness at display. In particular, color and tenderness are some of the most important purchasing criteria [2,3].

Meat discoloration results from the conversion of oxymyoglobin (MbO_2_) to metmyoglobin (MetMb) and an interaction between discoloration and lipid oxidation processes has been demonstrated [4]. Lipid oxidation is the major cause of deterioration in meat quality during storage and processing [5]. The primary and secondary oxidation products modify flavor, color, texture and decrease nutritional quality [6].

Lipid oxidation is a very complex process initiated by peroxidation of the unsaturated fatty acid in phospholipid membranes to form primary oxidation products, hydroperoxides. The hydroperoxides decompose into further secondary oxidation products, such as aldehydes, ketones, alkenes and alcohols that cause off-flavors and odors in meat, which negatively affect the acceptability and overall quality of meat and meat products [7].

The reactions responsible for myoglobin (Mb) and lipid oxidation generate products that can act mutually to accelerate oxidation [4]. Hence, antioxidants are added to maintain the quality and shelf-life of meat and other lipid-rich products. Antioxidants are capable of stabilizing free radicals by donating hydrogen (H) to free radicals, or accepting electrons from free radicals to form a complex [8]. Antioxidants are often lost during conversion of muscle to meat, processing and storage. Thus, adding them to the final product is one strategy used to minimize deterioration during storage and consequently increase the shelf-life of the product [7]. Synthetic antioxidants have been used extensively to minimize lipid oxidation in foods. However, due to an increasing concern about the safety of synthetic chemicals, the use of natural and bioactive compounds with antioxidant activity are preferred and have attracted the attention of researchers [9–11].

Essential oils (EOs) are natural compounds extracted from plants that exhibit antimicrobial and antioxidant properties, and therefore attract interest as additives in the food industry [12]. EOs from oregano (*Origanum vulgare* L.) and rosemary (*Rosmarinus officinalis* L.) have potential as natural food antioxidants [13–16]. Many EOs are considered to be ‘Generally Recognized as Safe’ (GRAS) and approved by the Food and Drug Administration (FDA) [17]. The use and choice of EOs should consider the consumer sensory acceptability of the final product. Indeed, due to their strong flavor, their direct use is often limited. To overcome this issue, EOs can be added into edible coatings, which have been suggested as an alternative food packaging to improve food safety and quality [18,19]. In beef display, polyvinyl chloride film (PVC) packaging is generally used, however, the exposure to oxygen accelerates oxidative processes and color degradation [20], resulting in a short shelf-life.

Edible coatings are typically formed using proteins and polysaccharides [21]. The salt derivative of alginic acid, alginate, is a natural anionic polysaccharide, composed of (1,4)-linked β-D-mannuronate and α-L-guluronate residues, isolated from brown algae [22]. Alginate can be cross-linked by the addition of divalent ions, such as Ca^2+^, to form strong gels and films. These films can be used to maintain the quality and prolong the shelf-life of foods by decreasing lipid oxidation, minimizing contact with oxygen, increasing water barrier properties and maintaining the flavor of the food [23]. Indeed, there is great interest in edible coatings due to their biocompatibility, biodegradability, broad application potential and use as carriers of functional ingredients [19]. Although limited research has been performed using edible coatings in red meats, their ability to maintain the quality of fish, chicken, fruits and vegetables, for example, has been well-demonstrated [24–31].

This study evaluated the effects of alginate-based edible coatings containing EOs (rosemary and oregano) on the quality, consumer acceptability and shelf-life of beef under refrigerated storage.

## 2. Materials and Methods

### 2.1. Material

Gallic acid, 2, 2′-azinobis-3-ethylbenzotiazoline-6-sulfonic acid (ABTS), 2, 2-diphenyl-1-picrylhydrazyl (DDPH), phosphate buffer, sodium carbonate and potassium persulfate were from Sigma-Aldrich (USA). Aluminum chloride, potassium ferricyanide and ferric chloride were from Vetec (Brazil). Calcium chloride was from Anidrol (Brazil), sodium alginate from Dinamica (Brazil) and EOs were from Ferquima (Brazil).

### 2.2. Preparation of samples

The meat was obtained from eight crossbred young bulls ½ Angus *vs*. ½ Nellore from a single father, finished in a feedlot for 187 days and slaughtered at 12-months old. The average live weight was 443.5 ± 26.2 kg. After slaughtering, carcasses were chilled at 4°C for 24 h. Then, the *Longissimus dorsi* (LD) was excised from the left half of the carcass from the seventh to the last lumbar vertebra. The LD was transported to the Laboratory of Animal Science, vacuum packaged and frozen intact at -18°C, until analysis (less than 1 month of storage).

Prior to analysis, the LD were thawed at 4°C for 24 h. Homogenous steaks of 2.5 cm thick were then obtained and distributed randomly for experimental treatment and analysis.

### 2.3. Preparation of the coating solutions and meat treatments

Sodium alginate solution was prepared by dissolving 20 g in 1 L of sterile distilled water at 70°C. After 30 min stirring to allow complete dissolution, the solution was chilled to 25°C. For active edible coating, the EOs were mixed with alginate solution (0.1%, defined by preliminary tests) under magnetic stirring. Steaks from each animal were randomly and equally divided into four groups: uncoated meat (CON); meat with edible coating (EC); edible coating with 0.1% rosemary EO (ECR) and edible coating with 0.1% oregano EO (ECO). Then, the steaks were individually submerged in alginate solution for 1 min, allowed to drain (to remove coating excess) for 1 min, submerged in calcium chloride solution (2% w/v) used as a crosslinking for 30 s and drained for a further 30 s. Each sample, with or without their respective edible coating, was packaged in an individual polystyrene tray over-wrapped with a retractile film (Goodyear®, Americana, São Paulo, Brazil) and stored refrigerated in an illuminated display at 2°C under light (fluorescent lamp, 1200 lux, 12 h day^-1^), simulating typical Brazilian market conditions. Samples of CON, EC, ECR and ECO were randomly removed at 1, 7 and 14 days of display (storage), for analysis.

### 2.4. Antioxidant activity

Antioxidant activity was assessed on the EOs (1:1000 v/v with methanol) to guarantee the antioxidant potential of the commercial product (the main components in its composition were: 1,8-cineol = 47.51%, camphor = 16.7% and α-pinene = 13.5% for rosemary oil; carvacrol = 70% for oregano oil). The three types of edible coatings were analyzed immediately after they were prepared (1:3 v/v with methanol) and also on the meat samples at 1 day of display (1:1 w/v with methanol), after extraction. Extracts were obtained by homogenization (in tubes homogenizer for oils and coating and ultra turrax for meat), centrifugation (15 min, 4.000 rpm) and for meat was also performed a filtration (filter paper). Antioxidant activity was assessed using the ferric reducing antioxidant power assay (FRAP), ABTS and DPPH assays.

#### 2.4.1. FRAP assay

The FRAP method was performed according to Zhu et al. [32]. Samples were mixed with methanol and an aliquot (250 μL) then mixed with 50 mM sodium phosphate buffer pH 7 (1.25 mL) and 1% potassium ferricyanide (1.25 mL) and incubated at 50°C for 20 min. Then, trichloroacetic acid (TCA) (10%) (1.25 mL) was added and the mixture was centrifuged at 3000 rpm for 10 min. The upper layer (2.5 mL) was mixed with 0.1% ferric chloride (500 μL) and the absorbance was measured at 700 nm. Results were expressed as mg gallic acid equivalent (GAE) g^-1^ oil, mg GAE g^-1^ coating and mg GAE 100 g^-1^ meat. The standard curve of gallic acid ranged from 0−300 mg L^-1^.

#### 2.4.2. ABTS radical scavenging assay

The ABTS assay was conducted according to Re et al. [33], with modifications. ABTS·^+^ was generated through the interaction of 7 mM ABTS (5 mL) with 140 mM potassium persulfate (88 μL). The mixture was incubated in the dark at 25°C for 16 h. The ABTS-activated radical was diluted with ethanol to an absorbance of 0.70 ± 0.02. The radical scavenging activity (%) was measured at 734 nm. Samples (40 μL) were mixed with ABTS·^+^ solution (1960 μL) and absorbance was recorded at 6 min. The radical scavenging activity (%) was calculated as:

$$ABTS\;radical\;scavenging\;activity\;(\%)=(1-(A_{sample\;t=0}/A_{sample\;t})*100$$

where: A _sample t = 0_: absorbance of the sample at time zero; A _sample t_: absorbance of the sample at 6 min.

#### 2.4.3. DPPH radical scavenging assay

DPPH scavenging activity was measured according to Li et al. [34], with modifications. Samples (150 μL) were mixed with 2850 μL of a methanolic solution containing DPPH (60 μM) and reacted for 30 min. The absorbance at 515 nm was measured against a blank of pure methanol. Antioxidant activity was calculated as:

$$DPPH\;radical\;scavenging\;activity\;(\%)=(1-(A_{sample\;t=0}/A_{sample\;t})*100$$

where: A _sample t = 0_: absorbance of the sample at time zero; A _sample t_: absorbance of the sample at 30 min.

### 2.5. Lipid oxidation

The malonaldehyde (MDA) content in meat was quantified using the thiobarbituric acid reactive substances (TBARS) assay [35]. The sample (5 g) was mixed with TCA solution (7.5% TCA, 0.1% EDTA and 0.1% gallic acid) (10 mL), homogenized using an Ultra Turrax, then centrifuged at 4°C for 15 min and 4.000 rpm. The supernatant was filtered and mixed with TBARS reagent (1% thiobarbituric acid, 562.5 μM, HCl, 15% TCA) (1:1 v/v). The mixture was boiled (100°C) for 15 min, cooled, then the absorbance measured at 535 nm against an MDA standard. Results were expressed as mg MDA kg^-1^ of meat. Lipid oxidation assays were performed at 1, 7 and 14 days of display.

### 2.6. Color

Color was evaluated by the CIELab system at 1, 7 and 14 days of display, using a Minolta CR-400 chromameter with a 10° view angle and a D65 illuminant. Six measurements at randomly selected points were recorded per sample, obtaining lightness (L*), redness (a*) and yellowness (b*). Chroma and hue values were calculated as follows:

$$Chroma=\sqrt{a{*}^{2}+b{*}^{2}}\;\text{and}\;Hue=\text{arctan}(b*/a*)$$

### 2.7. pH measurements

The pH was measured at 1, 7 and 14 days of storage, using a pH meter Text Model (Tradelab, Contagem, MG, Brazil) equipped with a penetration pH-electrode.

### 2.8. Water holding capacity

Water holding capacity (WHC) was determined as weight loss. The meat samples were removed from their trays at 1, 7 and 14 days of storage and their individual weights recorded on a semi-analytical scale. For each sample, results were expressed as a percentage of weight loss relative to its initial weight (day 0).

### 2.9. Shear force

Maximum shear force (N) was determined using a texturometer TA.XT Plus (Texture Technologies 15 Corp., UK), equipped with a Warner–Bratzler blade. The instrument was set with a 5 kg load cell and a crosshead speed of 1 mm/s. Six samples (1 cm × 1 cm × 2 cm) were prepared and each sheared once in the center and perpendicular to the longitudinal orientation of the muscle fibers.

### 2.10. Microstructure

The microstructure analysis was performed according to Matumoto-Pintro et al. [36], using a scanning electron microscope (SEM) (Superscan, Shimadzu SS-550) at 15 kV. Meat samples at 1 day of display were frozen-fixed in liquid nitrogen and lyophilized. Samples were mounted on aluminum stubs and coated with a gold layer (sputter coater, Bal-Tec, SCD 050).

### 2.11. Consumer acceptability

LD were thawed at 4°C for 24 h and homogenous steaks. The edible coatings were applied as described in section 2.3. Consumers only assessed acceptability on samples displayed for 1 day due to the Brazilian habit of consuming unaged meat. After 1 day, each steak was removed from the tray, covered with aluminum foil and cooked on a pre-heated grill (Grill Philco Jumbo Inox, Philco SA, Brazil) at 200°C until reaching an internal temperature of 70°C, monitored with a penetration thermocouple (Incoterm, 145 mm, Incoterm LTDA, Brazil). Each steak was cut into eight 2 x 2 cm cubes and kept warm (50°C) until consumer evaluation (less than 10 min after cooking).

Consumer testing was performed at the State University of Maringá (Brazil) in a private room adequately adapted to perform a sensory test. Ninety consumers were randomly selected among students, employees and visitors within quotas of gender (46 men and 44 women) and age (58.9% distributed from 18−24 years; 22.2% from 25−39 years; 7.8% from 40−54 years; and 11.1% > 55 years), according to the Brazilian national profile [37].

Nine sessions were carried out, each with ten different consumers. Each consumer evaluated four samples codified with a random three-digit code per session, corresponding to the different treatments (CON, EC, ECR and ECO). The meat was served in a randomized design to avoid order and carry-over effects [38]. Consumers were requested to taste and evaluate each sample on the acceptability of four attributes (odor, tenderness, flavor and overall acceptability) using a 9-point scale ranging from 1 = dislike extremely to 9 = like extremely. A medium level was not included, as described by Font i Furnols et al. [39]. Consumers were asked to eat unsalted toasted bread and rinse their mouth with water before evaluating each sample, including the first sample.

The sensory analysis protocol was previously approved by the Committee on Ethics in Research, at the State University of Maringa, PR, Brazil, under protocol CAAE48163215.4.0000.0104 and a written consent was obtained from participants in this study.

### 2.12. Statistical analyses

Meat attributes were assessed by analysis of variance using the general linear model (GLM) with SPSS (v.15.0) (IBM SPSS Statistics, SPSS Inc., Chicago, USA) for Windows. Means and standard deviation were calculated for each variable.

Type of edible coating (antioxidant activity) and display/storage time (lipid oxidation, pH, WHC, texture and color) were considered fixed factors in a factorial design, with three replicates per treatment for each analysis. When differences were statistically significant, a Tukey test was performed with statistical significance set at *p* = 0.05.

For consumer evaluation, the type of edible coating was considered a fixed factor and the consumer was considered a random factor, with nine replicates per treatment. Differences between means were evaluated using Tukey’s test (*p<*0.05). The correlations between attributes were evaluated using the Pearson correlation coefficient.

## 3. Results and Discussion

### 3.1. Antioxidant activity

The antioxidant activity can vary depending on the assay used due to differences in the underlying chemistry of the assay and the type of molecules detected, for example. Hence, at least two antioxidant methods are typically performed [40,41]. In this study, antioxidant activity was measured using the DPPH, ABTS and FRAP assays. The EO methanolic extracts had FRAP values of 3.56 and 0.38 mg GAE g^-1^ for oregano and rosemary, respectively. The ABTS and DPPH scavenging abilities (1:1000 w/v) for oregano were 69.94% and 27.31%, whilst these values were 15.79% and 4.08% for rosemary. Thus, all the assays used in this study demonstrated that the methanolic extract of oregano EO had a higher antioxidant activity (*p<*0.001) than rosemary EO (Table 1). This trend was also observed in the respective edible coatings and meat samples treated with the respective coatings (Table 1). The edible coatings with EO showed higher antioxidant activity (*p<*0.001) than CON and a similar trend was observed in the treated meat samples. Oregano EO showed the highest antioxidant activity in all analyses, whilst CON and EC had statistically similar antioxidant activity. Natural antioxidants have good potential in the meat industry as they are rich in active compounds and may minimize deterioration during storage [7,42]. The data showed that the EOs were responsible for the antioxidant activity in the coating and EC does not provide antioxidant activity.

**Table 1:** Radical scavenging activity (ABTS and DPPH radical scavenging) and ferric reducing power (FRAP) of essential oils, edible coatings and meat (with and without coating).

EO^1^				Rosemary	Oregano	*p*-value
	FRAP (mg GAE² g^-^^1^)			0.38±0.17	3.56±0.15	<0.001
	ABTS (%)			15.79±0.37	69.94±0.47	<0.001
	DPPH (%)			4.08±0.60	27.31±0.60	<0.001
Coating			EC	ECR	ECO	
	FRAP (mg GAE g^-^^1^)		n.d.*	n.d.*	n.d.*	-
	ABTS (%)		4.28±0.18^c^	8.56±0.56^b^	43.37±1.70^a^	<0.001
	DPPH (%)		4.08±1.21^c^	9.03±0.60^b^	18.92±0.32^a^	<0.001
Meat		CON	EC	ECR	ECO	
	FRAP(mg GAE 100g^-^^1^)	2.40±0.08^c^	2.61±0.04^c^	3.00±0.20^b^	3.61±0.26^a^	<0.001
	ABTS (%)	24.20±3.74^c^	26.11±1.02^c^	30.17±0.47^b^	35.38±3.74^a^	<0.001
	DPPH (%)	15.20±1.08^b^	15.80±1.60^b^	20.21±0.77^a^	20.58±1.08^a^	<0.001

Means with different lowercase letters in the same line are significantly different (*p<*0.05).

¹EO- Essential oil

²GAE–Gallic acid equivalent; CON–Meat without edible coating; EC–Coating and meat with edible coating without essential oil; ECR–Coating and meat with rosemary essential oil coating; ECO–Coating and meat with oregano essential oil coating.

*n.d.- no detected.

### 3.2. Lipid oxidation

The TBARS assay measures the secondary oxidation products responsible for oxidative rancidity [43]. The effect of the edible coatings on oxidative stability of the meat was evaluated throughout storage. The inclusion of the EO in coating and storage time significantly affected TBARS values (Table 2). Lipid oxidation increased significantly (*p<*0.001) during storage, particularly in the control sample (CON), which showed the highest increase. The EO coatings decreased lipid oxidation compared to CON and EC. ECO was more effective than ECR. At 14 days, the TBARS values reached approximately 1.00, 0.91, 0.61 and 0.53 mg MDA kg^-1^ for CON, EC, ECR and ECO, respectively, corresponding to a lipid oxidation decrease of approximately 47 and 39% for ECO and ECR respectively. This difference between oregano and rosemary could be due to the different chemical composition of their EOs. Rosemary has no major antioxidant component with presumable high antioxidant activity (like phenols). On the other hand, oregano has the carvacrol (terpenoid family) as the main component comprising phenolic compounds, acting as antioxidants due to their high reactivity with peroxyl radicals, which are eliminated by H-donation [44,45]. Oxidation is the main non-microbial factor responsible for the quality deterioration in foods and is one of the major reasons for consumer rejection and loss of quality during storage [46]. Lipid oxidative stability depends on the balance between antioxidant and pro-oxidant components [47]. This study shows that the use of natural antioxidant sources could be effective at preventing lipid oxidation in meat products during refrigerated storage. Cardoso et al. [48] found that in beef with an edible chitosan gelatin-based coating, low TBARS values were related, in part, to chitosan and its antioxidant property. Raeisi et al. [49] found lower TBARS values for coated (carboxymethylcellulose-based coatings incorporated with *Zataria multiflora* Boiss EO and grape seed extract) rainbow trout fillets compared to uncoated samples during the shelf-life of the product and associated this decrease to the synergism between the strong antioxidant activity of the natural ingredients and the oxygen barrier properties of the coating. Volpe et al. [24] found that a carrageenan coating, both with and without lemon EO, limited lipid oxidation of stored fresh trout fillets. Song et al. [23] showed that refrigerated bream had lower TBARS values when coated with a sodium alginate-based edible coating with natural antioxidants (vitamin C and tea polyphenols) than the uncoated samples, and although the coating effectively improved the quality and shelf-life of the product by decreasing lipid oxidation, this was further improved by the antioxidants in the coating. Thus, an edible coating incorporating a natural antioxidant, such as EO, may improve the shelf-life of meat products by preventing its lipid oxidation.

**Table 2:** Effect of active edible coating on lipid oxidation (TBARS) expressed as mg malonaldehyde kg^-1^ of meat during storage at 2°C.

Storage (days)	CON	EC	ECR	ECO	*p*-value
1	0.39±0.01^aC^	0.36±0.06^aC^	0.29±0.02^bC^	0.25±0.01^bC^	0.007
7	0.69±0.03^aB^	0.61±0.01^bB^	0.46±0.01^cB^	0.41±0.02^dB^	<0.001
14	1.00±0.02^aA^	0.91±0.01^bA^	0.61±0.07^cA^	0.53±0.02^dA^	<0.001
*p*-value	<0.001	<0.001	<0.001	<0.001	

Means with different lowercase letters in the same line are significantly different (*p*<0.05). Means with different uppercase letters in the same column are significantly different (*p*<0.05). CON–Control without edible coating; EC–Meat with edible coating; ECR–Meat with edible coating and rosemary essential oil; ECO–Meat with edible coating and oregano essential oil.

### 3.3. Color

The optical properties of an edible coating can change the overall appearance of food, since coating color may vary depending on the type of material used for their production. Moreover, in relation to meat, the typical form of Myoglobin, the principal protein responsible for meat color, associated with low oxygen concentration (deoxymyoglobin—with edible coating) or with oxygenation (oxymyoglobin—without edible coating) can influence meat purchasing decisions [2], so it was important to compare the color of the meat with and without an edible coating. Color values (L*, a*, and b*) for all treatments as a function of storage time are provided in Table 3. The L*, or lightness values, decreased with time for all treatments. CON presented a higher L* value, possibly caused by changes in the meat structure related to the highly oxidizing conditions, such as protein conformational changes, which may increase light dispersion [50]. The committed structural and conformational stability of proteins caused by oxidative damage may result in rupture of the peptide sequence, interactions (protein–protein such as formation or polymerization of aggregates) and modification of the amino acid chains. These modifications caused by oxidative process may alter protein function and its structure. From the most relevant chemical modifications, the formation of protein cross-links and protein carbonylation have been associated with the muscle protein functionality losses and modifications of meat attributes as color, flavor and texture [51]. Also, the maintenance of exudates in the coated meat (Table 4) darkens the color. Łopacka et al. [52] also observed an increase in lightness when comparing packaging systems with high oxygen contact in relation to the vacuum. Regarding a* or redness values, CON showed a significant decrease (*p<*0.001) during storage. The coating significantly decreased (*p<*0.05) the color losses compared to CON. Indeed, at 14 days of storage, the a* values of coated meat remained >10, indicating a bright red color [53]. Meat pigment, in the absence of oxygen, is in the form of deoxy or reduced Mb, which has a purple-red color. On air exposure, the pigment is oxygenated to form MbO_2_, conferring a bright red color to the meat [54]. The coating slows down the oxygenation process, therefore, instead of reaching the maximum a* value after the first days of blooming due to MbO_2_ formation, this maximum value is reached at approximately 7 days, decreasing thereafter. There was a significant difference (*p<*0.001) between the b* values of the control and coated samples and CON showed a more pronounced decrease in b* value during storage. Coated meat exhibited the highest b* values, which could be attributed to the yellowish color of the coating. No significant differences were found among the coated treatments. Colors that exhibit low chroma values are considered pale [48] and in this study, CON showed a lower chroma value than coated meat throughout storage, which may not be appealing to consumers at purchase time. Fresh meat typically becomes less red and lighter after a few days. Thus, an edible coating that can maintain redness and intensify the meat color could lead to an extension in meat color display-life [48]. The hue value (H°) showed a significant difference (*p<*0.001) between treatments. At day 1, CON had the lowest H°. During storage, however, its H° increased, while the H° of coated meats decreased at day 7 and then increased at day 14, to give a final H° value similar to day 1, indicating minimal color deterioration. Cardoso et al. [48] observed similar behavior in meat with a chitosan gelatin-based edible coating, which exhibited a darker color (more intense chroma and reddish hue) compared to the control sample. Fresh beef is susceptible to fast deterioration due to the high level of protein and moisture. Thus, food industries are looking for new alternatives to extend its shelf-life. The edible coating with natural additives such as essential oils can reduce or inhibit degradative processes during the display, besides add flavor to the product.

**Table 3:** L*a*b*, chroma and hue values of meat with and without an edible coating during storage.

	Storage (days)	CON	EC	ECR	ECO	*p-v*alue
L*						
	1	40.22±2.11^Aa^	39.38±1.21^Aab^	38.53±1.68^Aab^	37.30±1.55^Ab^	0.012
	7	38.74±1.82^Aa^	34.62±1.19^Bb^	36.13±1.19^Bb^	36.53±2.63^ABb^	<0.001
	14	36.23±1.56^Ba^	33.61±2.33^Bb^	34.24±1.46^Bab^	34.85±2.06^Bab^	0.043
	*p*-value	<0.001	<0.001	<0.001	0.020	
a*						
	1	13.25±1.36^Ab^	15.71±1.77^Ba^	15.01±0.92^Ba^	16.32±2.18^Ba^	0.001
	7	11.24±0.51^Bb^	17.88±1.43^Aa^	17.05±1.42^Aa^	18.30±1.28^Aa^	<0.001
	14	8.29±0.87^Cb^	15.15±1.26^Ba^	15.72±1.77^ABa^	16.32±1.91^ABa^	<0.001
	*p*-value	<0.001	0.001	0.011	0.001	
b*						
	1	14.07±1.04^Ab^	18.69±0.99^Aa^	17.43±1.54^ABa^	17.67±0.53^ABa^	<0.001
	7	12.71±0.54^Bb^	18.67±0.65^Aa^	18.75±0.74^Aa^	18.63±0.59^Aa^	<0.001
	14	10.70±0.43^Cb^	16.59±1.28^Ba^	17.08±1.25^Ba^	17.48±1.30^Ba^	<0.001
	*p*-value	<0.001	<0.001	0.012	0.017	
Chroma						
	1	19.34±1.56^Ab^	24.83±2.90^Aa^	22.96±1.68^Ba^	24.85±0.84^ABa^	<0.001
	7	16.94±0.74^Bc^	25.57±0.47^Aa^	25.56±1.61^Aa^	26.26±0.80^Aa^	<0.001
	14	13.60±0.79^Cc^	22.34±1.15^Bb^	24.22±1.67^ABa^	23.59±2.19^Bab^	<0.001
	*p*-value	<0.001	0.001	0.006	0.001	
Hue						
	1	44.04±0.68^Cc^	46.45±0.99^Ab^	48.43±1.86^Aa^	46.14±2.37^Ab^	<0.001
	7	49.22±1.30^Ba^	40.66±1.81^Bc^	43.90±2.51^Bb^	41.95±1.48^Bbc^	<0.001
	14	52.98±1.73^Aa^	45.43±2.62^Ab^	46.64±1.46^Ab^	45.66±2.64^Ab^	<0.001
	*p*-value	<0.001	<0.001	<0.001	<0.001	

Means with different lowercase letters in the same line are significantly different (*p<*0.05). Means with different uppercase letters in the same column are significantly different (*p<*0.05). CON–Control without edible coating; EC–Meat with edible coating; ECR–Meat with edible coating and rosemary essential oil; ECO–Meat with edible coating and oregano essential oil.

**Table 4:** pH, weight loss and shear force of meat samples (with and without edible coating) during cold storage.

	Storage (days)	CON	EC	ECR	ECO	*p*-value
pH						
	1	5.71±0.13^a^	5.97±0.08^b^	5.99±0.03^b^	6.03±0.05^b^	0.007
	7	5.73±0.08^a^	5.89±0.01^b^	5.96±0.05^b^	5.95±0.04^b^	0.004
	14	5.74±0.14^a^	6.06±0.12^b^	6.05±0.10^b^	6.06±0.01^b^	0.016
	*p*-value	0.961	0.137	0.328	0.054	
Weight loss (%)						
	1	3.35±0.57^aB^	1.84±0.05^bC^	1.45±0.07^bB^	1.87±0.08^bB^	<0.001
	7	8.88±0.50^aA^	6.26±0.46^bB^	6.33±0.71^bA^	6.02±0.76^bA^	0.002
	14	9.27±0.22^aA^	7.96±0.32^bA^	7.07±0.55^bA^	7.71±0.97^bA^	0.011
	*p*-value	<0.001	<0.001	<0.001	<0.001	
Shear force (N)						
	1	6.18±0.31^aA^	5.43±0.42^bA^	5.39±0.34^bA^	5.19±0.59^bA^	0.004
	7	5.62±0.15^aB^	4.75±0.43^bB^	4.30±0.62^bB^	4.11±0.33^bB^	<0.001
	14	5.20±0.10^aC^	3.64±0.40^bC^	3.43±0.36^bC^	3.47±0.20^bC^	<0.001
	*p*-value	<0.001	<0.001	<0.001	<0.001	

Means with different lowercase letters in the same line are significantly different (*p<*0.05). Means with different uppercase letters in the same column are significantly different (*p<*0.05). CON–Control without edible coating; EC–Meat with edible coating; ECR–Meat with edible coating and rosemary essential oil; ECO–Meat with edible coating and oregano essential oil.

### 3.4. pH measurement, weight loss analysis and shear force of meat

The pH values, weight loss and shear force of meat during storage are presented in Table 4. There were no significant differences in pH values (*p*>0.05) among the coated samples. However, differences were found between the CON and coated meats. EC, ECO and ECR had a higher pH value than CON, probably due to the coating pH (approximate pH 6).

The edible coating significantly decreased weight loss in the meat samples during all days of display. Little or no exudate from the coated samples was visible, which is an important attribute as its presence is not attractive to the consumer [55]. Weight loss progressively increased for all samples during storage, ranging from 3.35−9.27, 1.84−7.96, 1.45−7.07 and 1.87−7.71% for CON, EC, ECR and ECO, respectively.

The shear force decreased significantly with storage for all samples (*p<*0.001), although less intense in CON, which had the highest shear force throughout storage (Table 4). This difference in tenderness may be related to the WHC of the coating, maintaining the water within the system and providing a juicier and more tender beef. Also, higher oxygen concentrations in contact with uncoated meat (CON) may increase oxidation levels of lipids and proteins. One consequence of protein oxidation is protein aggregation, which can occur in various ways. Oxidation of cysteine thiol groups to form disulfide bonds, reaction between a carbonyl and a protein, and cross-linking between the proteins through the amino groups of lysine residues, can all lead to protein aggregation. Moreover, cross-links formed in highly oxidative conditions can decrease protein susceptibility to proteolytic enzymes that decrease tenderness by proteolysis [56–58]. Some authors evaluated various packaging during meat storage and found a higher tenderness in samples that had less contact with oxygen [58,59].

### 3.5. Microstructure

**Fig 1:**
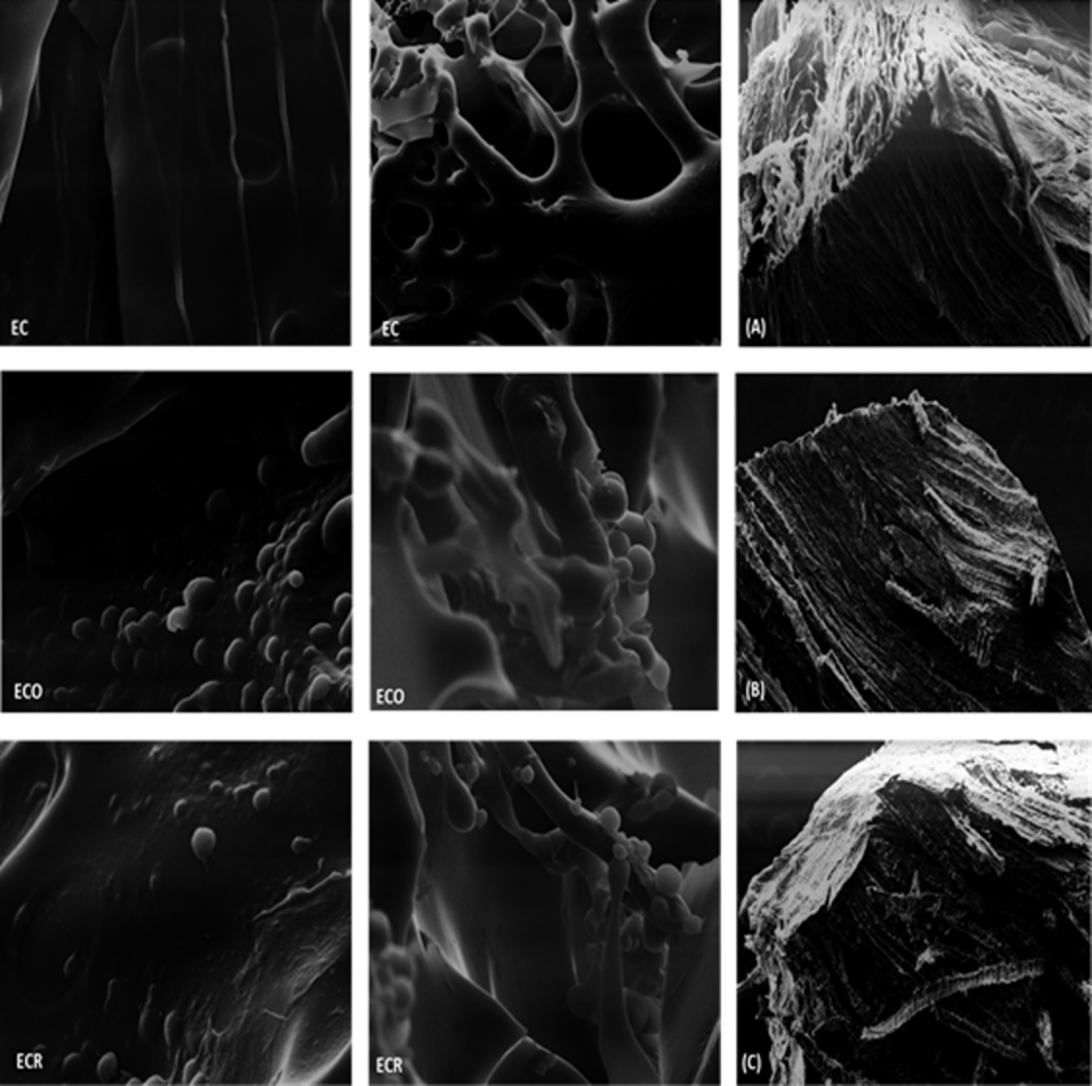
Scanning electron micrographs of the coating without oil (EC), with oregano essential oil (ECO), with rosemary essential oil (ECR), alginate coating (A) and meat with alginate coating (B and C). Magnification of 40x, 1200x, 1800x.

The microstructure was examined to verify the droplet organization within the biopolymer matrix of the EO edible coatings, and gain insight into its influence on the coating properties. Meat and coating microstructures were observed (Fig 1). The coating without EO was smooth and homogeneous (Fig 1 –EC with 1200 and 1800x magnification). In contrast, the ECO and ECR exhibited similar, heterogeneous structures, with oil droplets dispersed throughout the polymer matrix. Similar features have been observed in previous studies on edible films/coatings with EOs [18,60,61]. Fig 1B and 1C) at a comparable magnification (40x) show the coating layer extends throughout the meat both horizontally and vertically, respectively and Fig 1A (40x) shows the structure of edible coating on its own.

### 3.6. Consumer acceptability

ECO samples had a significant effect on consumer perception for odor (*p*<0.019), flavor (*p*<0.001) and overall acceptability (*p*<0.011). In particular, ECO had higher odor acceptability than CON and EC. ECR showed intermediary odor values among all the samples. Tenderness acceptability did not vary between treatments (Table 5).

**Table 5:** Consumer (n = 90) acceptability^§^ of coated and uncoated meat.

	CON	EC	ECR	ECO	*p*-value
Odor	6.29±1.86^b^	6.36±1.17^b^	6.46±1.83^ab^	6.91±1.57^a^	0.019
Tenderness	6.68±1.82	6.87±1.69	7.02±1.52	6.99±1.41	0.304
Flavor	6.49±1.90^ab^	6.47±1.65^ab^	5.99±2.11^b^	6.94±1.67^a^	0.001
Overall acceptance	6.46±1.88^ab^	6.57±1.59^ab^	6.30±1.85^b^	6.98±1.46^a^	0.011

Means with different lowercase in the same line are significantly different (*p<*0.05). CON–Control without edible coating; EC–Meat with edible coating; ECR–Meat with edible coating and rosemary essential oil; ECO–Meat with edible coating and oregano essential oil.

^§^Based on a 9-point scale (1: dislike extremely; 9: like extremely).

EOs have unique aromatic profiles. Traditionally, particular herbs or spices are associated with certain meals. Both, rosemary and oregano are meat spices. However, according to the acceptability results, oregano was more appreciated, with higher acceptance scores, than rosemary.

Edible coatings create a gelatinous layer around the meat, which adheres to the meat after cooking. It might be possible that consumers detected the consequent change in meat texture and reported a difference in acceptability from CON. However, the consumers did not notice this tenderness variation. Despite CON showing a significantly higher shear force from all the other samples at day 1, meat from the four treatments could be considered as tender and consumers did not notice those variations. Also, the presence of an edible coating did not affect acceptability. Nevertheless, the high acceptability after 1 day of aging reflects the particular culinary background of Brazilian consumers.

The addition of EO within the edible coating modified flavor acceptability. Compared to the samples without EO (CON and EC) this acceptability varied depending on the type of oil used. Oregano showed significant higher acceptability values than rosemary. Nevertheless, ECR had similar acceptance values to the samples coated without EOs. Consumers preference for a product is typically associated with their previous personal and culinary background [62]. Food preferences are developed in infancy and early childhood, affecting individual food preferences later in life [63].

In typical consumer tests, meat tenderness is one of the main variables that influences overall acceptability [64]. However, in the current study, the correlation between overall acceptability and tenderness was lower (r = 0.645) than the other attributes, probably because it became a lower priority than the other attributes, which could be attributed to the EOs. Indeed, both flavor (r = 0.882) and odor (r = 0.723) were highly correlated with the overall acceptability of the samples.

## 4. Conclusions

The alginate-based edible coatings effectively decreased the weight loss and lipid oxidation in the meat for up to 7 and 14 days of retail display, respectively. Coated meat was redder, had a more intense chroma and was more tender. The inclusion of EOs in the coating increased the antioxidant activity. Compared to ECR, ECO showed higher antioxidant activity, lower lipid oxidation and higher consumer acceptance. Thus, edible coatings containing EO have potential application in animal meat products to maintain/improve their characteristics during the shelf-life.
